# Sensitivity of virtual non-contrast dual-energy CT urogram for detection of urinary calculi: a systematic review and meta-analysis

**DOI:** 10.1007/s00330-022-08939-5

**Published:** 2022-06-28

**Authors:** Katherine McCoombe, Karen Dobeli, Steven Meikle, Stacey Llewellyn, Peter Kench

**Affiliations:** 1 Faculty of Medicine and Health, University of Sydney, Camperdown, New South Wales Australia; 2 Department of Medical Imaging, Royal Brisbane and Women’s Hospital, Herston, Queensland Australia; 3 QIMR Berghofer Medical Research Institute, Herston, Queensland Australia

**Keywords:** Dual-energy CT, Urogram, Urinary calculi, Sensitivity

## Abstract

**Objective:**

To determine the sensitivity of dual-energy (DE) virtual non-contrast computed tomography (vNCT), generated from the excretory phase of a CT urogram, compared to true non-contrast CT (tNCT) for the detection of urinary calculi.

**Methods:**

A search of multiple medical literature databases was performed using predetermined search terms. Inclusion and exclusion criteria were applied, and bias risk was assessed by two independent reviewers using the quality assessment of diagnostic accuracy studies (QUADAS) tool. Collated estimates of sensitivity were generated, and sources of heterogeneity were identified and reviewed.

**Results:**

Thirteen studies (1760 patients; 1740 urinary calculi) were included for sensitivity assessment. Pooled sensitivity for urinary calculi on vNCT was 78.1% (95% CI: 70.2 to 85.0%); however, heterogeneity between studies was very high (*I*^2^ = 92.0%). Sources of heterogeneity between studies were explored through subgroup analysis by categorising studies according to slice thickness (≥ 2 mm and < 2 mm), use of oral hydration, and use of intravenous furosemide. Pooled sensitivity for detection of urinary calculi on vNCT for studies that used oral hydration and < 2 mm slice thickness was 92.2% (95% CI: 89.5 to 94.5%). Pooled specificity was not performed as true negatives were not reported in most studies. Potential sources of bias were identified in included studies.

**Conclusion:**

vNCT demonstrated a moderate pooled sensitivity compared to tNCT for the detection of urinary calculi in split bolus CT urogram protocols. However, subgroup analysis suggests higher sensitivity when employing oral hydration and < 2 mm slice thickness or increment.

**Key Points:**

• *vNCT demonstrated moderate pooled sensitivity for the detection of urinary calculi in split bolus CT urogram protocols*.

• *Subgroup analysis suggested higher sensitivity with oral hydration and < 2 mm slice thickness or increment*.

**Supplementary Information:**

The online version contains supplementary material available at 10.1007/s00330-022-08939-5.

## Introduction

Haematuria is a common finding in clinical practice and can be described as visible or microscopic [1]. The prevalence of microscopic haematuria varies from 1 to 18%, depending on patient age, gender, and rate of testing. Clinical differentials for microscopic haematuria are urinary calculi, malignancy, and strictures [2]. Urolithiasis is particularly common, affecting the Australian population with an incidence of 0.13% per year [3]. The lifetime prevalence of urinary calculi is higher for males (15%) compared to females (8%) [3].

Computed tomography (CT) is the gold standard imaging test for microscopic haematuria as it can diagnose urinary calculi, renal masses, and urothelial tumours [4]. CT urography has a reported low diagnostic yield of 22.1% for clinically significant cause of haematuria [5, 6]. A contemporary multi-detector computed tomography (MDCT) urogram protocol consists of a non-contrast scan and one or two subsequent acquisitions after the intravenous administration of iodinated contrast. The true non-contrast CT scan (tNCT) is performed to identify urinary calculi [4]. Bhojani et al [7] reported that tNCT alone has a sensitivity of 95–100% for the detection of urinary calculi. Either combined (dual phase) or separate nephrographic and late excretory phase acquisitions are then performed to identify renal masses and urothelial tumours.

A disadvantage of CT urography is the radiation dose, which can be up to three times that of a routine CT abdomen scan due to the multiple phases required [2]. Advances in CT technology have given rise to the development of dual-energy (DE) and spectral MDCT. This technology enables advanced CT data postprocessing, such as the subtraction of the iodine attenuation from contrast-enhanced acquisitions to produce a virtual non-contrast CT (vNCT) reconstruction.

The ability to generate a vNCT reconstruction from a contrast phase scan may enable replacement of the tNCT scan, therefore reducing the radiation dose during CT urography. The radiation dose savings vary between studies that use a single-energy or dual-energy split-bolus CT urogram protocol. Manoharan et al [2] using a split bolus dual-energy tNCT protocol reported a radiation dose saving for dose length product (DLP), CT dose index (CTDI), and effective dose as 47.5%, 48.2%, and 47.9% respectively. Karlo et al [8] using a split bolus single-energy tNCT reported a radiation dose reduction of 28 ± 6%.

Oral hydration, intravenous (IV) hydration (> 250 ml), and IV diuretics are all techniques documented to aid complete opacification of the renal collecting systems on excretory phase acquisition [9, 10]. Thinner slices (< 2 mm) are also documented to improve detection of urinary calculi on tNCT [11].

A number of studies have been undertaken since 2010 to investigate the potential for vNCT to replace the tNCT phase in DECT urography. The purpose of this systematic review and meta-analysis is to determine the sensitivity of vNCT, generated from the excretory phase of a CT urogram, compared to tNCT for the detection of urinary calculi.

## Methods

### Search strategy

**Fig. 1:**
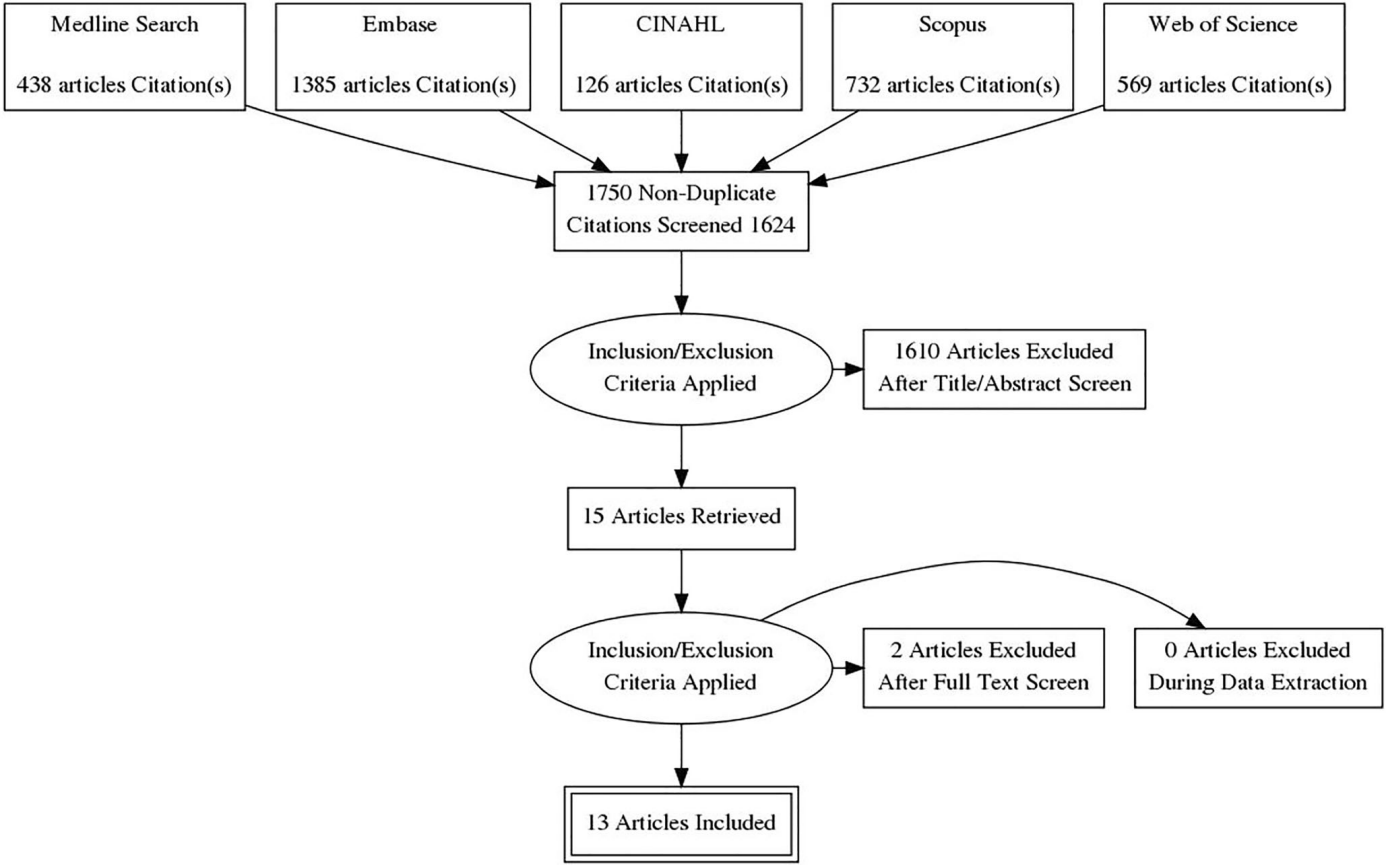
Flow diagram illustrating search results, study review, and study inclusions and exclusions

An electronic literature search using the databases Medline, SCOPUS, Embase, CINHAL, and Web of Science was performed for articles published between 1 January 2006 and 6 May 2021. The Preferred Reporting Items for Systematic reviews and Meta-Analysis (PRISMA) methodology was used to conduct this systematic review and meta-analysis (Fig. 1).

The following PICO question was proposed: *p*atients with haematuria; *i*ndex test of a vNCT generated from the excretory phase of a DECT urogram study used for diagnosis; in *c*omparison to the reference standard of tNCT performed on the same patients. Extracted data were used to compare the *o*utcome, which was sensitivity of the detection of urinary calculi.

To ensure a thorough search of the databases, all synonyms, abbreviations, and common adjectives were searched. Table 1 lists the searched terms and algorithms. The reference list of all resulting articles was also reviewed to identify relevant articles not identified through the database searches. DECT became commercially available (FDA approved) in 2006; thus, all articles prior to this date were excluded [12].

**Table 1:** Algorithm and keywords used as search terms

CT DECT Spectral CT	OR	Computed Tomography Dual energy computed tomography
AND		
Urography Intravenous pyelogram	OR	IVP Urogram
AND		
Urinary Calculi Urolithiasis Kidney Calculi Ureteral Calculi Renal stone Renal tract calculi	OR	Renal tract calculus Nephrolithiasis Kidney Calculus Ureteral Calculus Stone Calculi

### Inclusion criteria

1. Human patients undergoing CT urogram
2. Dual-energy/spectral CT was performed and a vNCT series was generated as part of the protocol.
3. The data retrieved was sufficient to calculate the sensitivity.
4. True NCT was performed as the reference test.
5. Peer-reviewed journal articles.
6. English language article.

### Data extraction

Data extraction was performed by one author (KM). Data extraction included the study’s first author, year of publication, country of origin, study centre, number of patients, patient age (mean, range), technical parameters of dual-energy CT imaging (manufacturer and dual-energy CT technique), CT protocol (contrast volume and phases performed), method of urine dilution (IV hydration, oral hydration, and IV diuretics), and number of reported true positives (TP), false positives (FP), and false negatives (FN).

The following definitions were used: TP is a positive index test (vNCT) where diagnosis of renal calculus is confirmed by the reference standard (tNCT). FP is a positive index test where diagnosis of renal calculus is rejected by the reference standard. FN is a negative index test where diagnosis of renal calculus is made on the reference standard.

### Quality assessment

Two authors (K.M. and K.D.) reviewed the included studies and independently applied a customised Quality Assessment of Diagnostic Accuracy Studies (QUADAS) tool [13] (Table 2). The 12 QUADAS criteria described in Table 2 were marked as ‘yes’, ‘no’, or ‘unclear’. The four key areas of focus were patient selection, reference standard, index test, and review bias. Disagreements were resolved by consensus discussion.

**Table 2:**
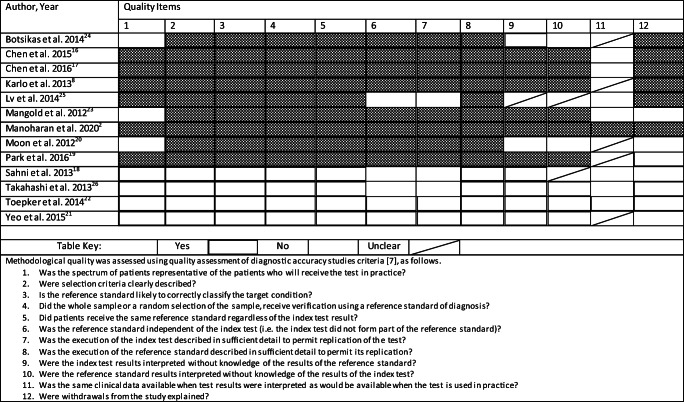
Results of QUADAS tool assessment for risk of bias assessment

### Data analysis

Data was presented in all included studies on a per-calculus rather than per-patient or per-segmental basis. Pooling of data to generate combined estimates was performed for sensitivity only which was defined as TP/(TP + FN). Pooled specificity analysis was not able to be performed as TN results were not reported in any of the included studies for per-calculus level (refer to Table 4).

Meta-analysis was performed to calculate the pooled sensitivity and 95% confidence intervals (CI) using a random-effects model with DerSimonian and Laird weights. The Freeman-Tukey double arcsine transformation of the raw proportions was employed to stabilise the variances and allow appropriate use of normal approximation procedures for proportions close to 100% [25].

Heterogeneity, in the form of between-studies variation, was measured using the *I*^2^ index, estimated by an inverse variance fixed-effects model using the levels as defined by Higgins et al [26] (low < 25%, moderate 25–75%, and high > 75%). Sources of heterogeneity were explored including the effect on sensitivity. Subgroups based on common parameters known to affect renal calculus detection and urography image quality were evaluated based on the combined occurrence: slice thickness, use of oral and IV hydration, and administration of furosemide. Publication bias was assessed using a funnel plot. Meta-analysis was performed in Stata Statistical Software: Release 15 using Metaprop [25].

## Results

### Eligible studies and quality assessment

Figure 1 illustrates the PRISMA flow diagram used for study eligibility. The search strategy identified 3374 articles. From these, 1750 were found to be duplicates and subsequently excluded. After the title and abstract of the remaining 1624 articles were analysed, 1610 were removed based on the described inclusion and exclusion criteria. The full text was analysed for the remaining 15 articles. Two of the 15 articles were not written in English and therefore excluded. The reference list of all review articles was also checked manually for additional articles. No additional suitable publications for inclusion were identified. Two articles by the same author were both included as the data collection periods for these studies did not overlap [15, 16].

Thirty-two (32) of the 132 QUADAS assessments across the remaining 12 studies were classed as ‘no’ or ‘unclear’. No studies were excluded from the meta-analysis based on the QUADAS tool assessment (Table 2).

### Data extraction

Table 3 summarises the characteristics of the included studies. Appendix 1 summarises the data extracted about the different technical parameters of each of the studies. Table 4 summarises the data extracted from the studies regarding sensitivity of the detection of urinary calculi.

**Table 3:** Characteristics of included studies

Literature (author and year)	Patients					Study centre
	No. of included patients	Mean Age	Gender		Number of urinary calculi	
			Male	Female		
Botsikas et al 2014 [14]	116	70.2	92	24	23	Geneva University Hospital, Geneva, Switzerland
Chen et al 2015 [15]	84	57.7	46	38	32	Graduate Institute of Medicine, College of Medicine, Kaohsiung Medical University, Taiwan
Chen et al 2016 [16]	171	59.8	99	72	45	Graduate Institute of Medicine, College of Medicine, Kaohsiung Medical University, Taiwan
Karlo et al 2013 [8]	100	47	59	41	104	University Hospital Zurich, Zurich, Switzerland
Lv et al 2014 [17]	46	51	33	13	136	The First Affiliated Hospital of Zhengzhou University, Guangzhou, China
Mangold et al 2012 [18]	152	60.3	97	55	87	University of Tübingen, Tübingen, Germany
Manoharan et al 2020 [2]	130	40	78	52	129	All India Institute of Medical Science, Ansari Nagar, New Delhi, India
Moon et al 2012 [19]	146	56	73	73	80	Samsung Medical Centre, Sungkyunkwan University School of Medicine, Seoul, South Korea
Park et al 2016 [20]	296	57.9	167	129	148	Samsung Medical Centre, Sungkyunkwan University School of Medicine, Seoul, South Korea
Sahni et al 2013 [21]	100	61	55	45	64	Brigham and Women’s Hospital, Harvard Medical School, Boston, USA
Takahashi et al 2013 [22]	62	66.1	38	24	43	Department of Radiology, Mayo Clinic, Rochester, USA
Toepker et al 2014 [23]	81	51	47	34	350	Medical University Vienna, Vienna General Hospital, Vienna, Austria
Yeo et al 2015 [24]	356	41.8	211	145	499	Dongsan Hospital, Keimyung Hospital, Kyungpook National University Hospital, Daegu, South Korea

**Table 4:** Results of studies included in pooled sensitivity analysis

Literature (author and year)	Number of urinary calculi true non-contrast CT	Virtual non-contrast CT						Slice thickness × increment	Patient preparation (IV, oral hydration, furosemide, none)
		TP	FP	FN	Sensitivity (per calculus)	Specificity (per patient)	Diagnostic accuracy		
Botsikas et al 2014 [14]	23	18	0	5	78.2%	NA	NA	2 mm × 2 mm	Furosemide
Chen et al 2015 [15]	32	28	3	4	87.5%	94.6%	92.1%	1.5 mm × 1 mm	Oral
Chen et al 2016 [16]	45	39	4	6	86.7%	98.5%	95.5%	1.5 mm × 1 mm	Oral
Karlo et al 2013 [8]	104	86	0	18	83.0%	NA	83.0%*	2 mm × 1.6 mm	None
Lv et al 2014 [17]	136	101	0	35	74.3%	NA	NA	0.625 mm × 0.625 mm	None
Mangold et al 2012 [18]	87	46	0	41	52.9%	NA	NA	2 mm × 1 mm	None
Manoharan et al 2020 [2]	129	118	0	11	91.5%	NA	91.5%*	1.5 mm × 1.5 mm	Oral
Moon et al 2012 [19]	80	59	0	21	73.8%	NA	NA	1.5 mm × 3 mm	Furosemide
Park et al 2016 [20]	148	100	0	48	67.6%	NA	NA	1.5 mm × 3 mm	Oral + furosemide
Sahni et al 2013 [21]	64	42	0	22	65.6%	NA	NA	3 mm × 3 mm	Oral + furosemide
Takahashi et al 2013 [22]	43	27	0	16	63%	NA	NA	1.5 mm × 1.5 mm	None
Toepker et al 2014 [23]	350	289	13	61	82.6%	100%	98.8%	1 mm × 0.8 mm	None
Yeo et al 2015 [24]	537	499	0	38	95.1%	NA	92.9%	1.5 mm × 1.5 mm	Oral

*NA* not applicable (required data not provided in the article)

*Diagnostic accuracy (per calculus) reported as sensitivity

### Data analysis

**Fig. 2:**
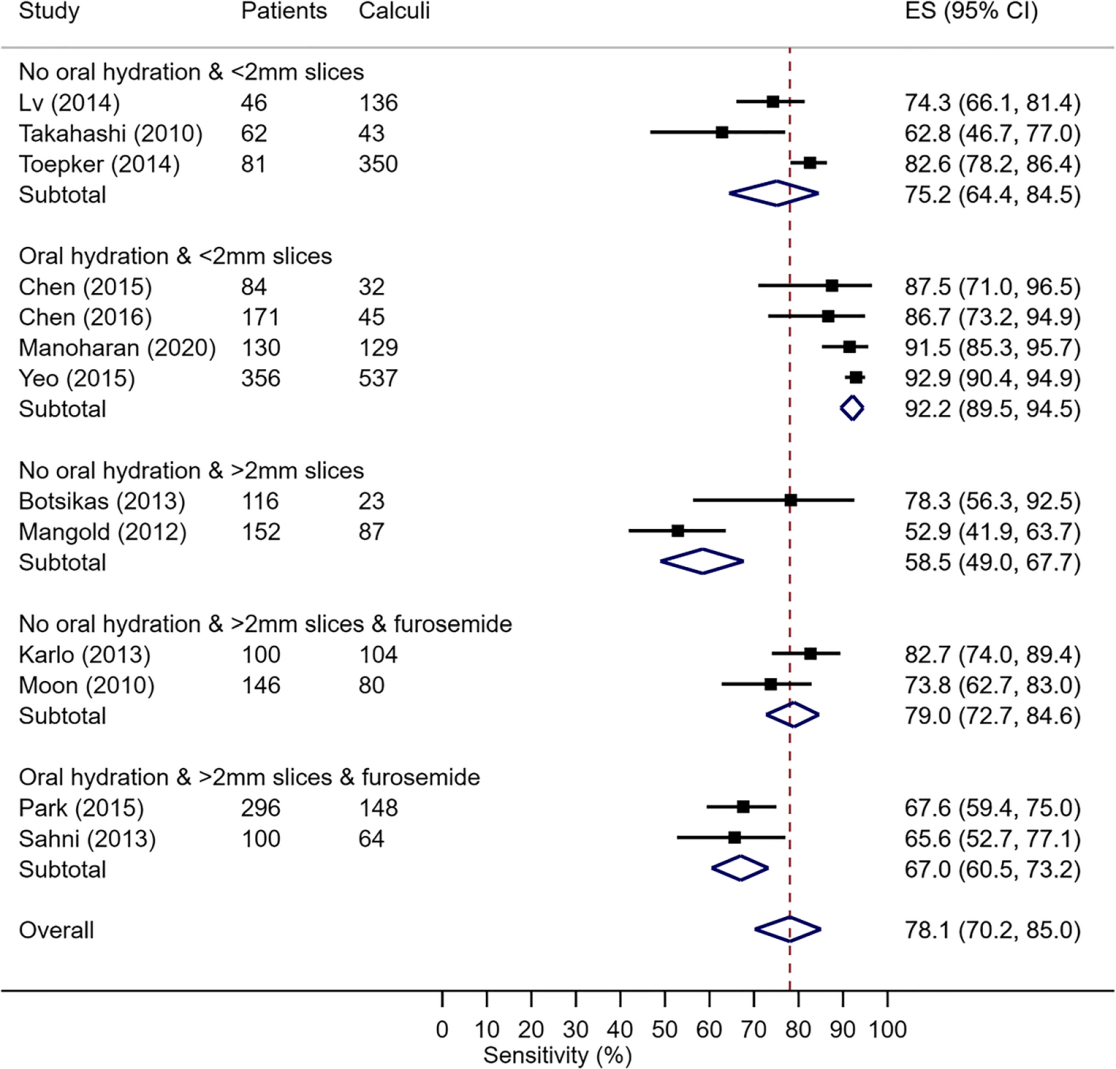
Forest plot of sensitivity for vNCT for the diagnosis of urinary calculi. Included studies are listed on the left with the summary of accuracy measurements, 95% CI, and pooled sensitivity listed on the right.

Figure 2 shows a forest plot of the pooled sensitivity data for the diagnosis of urinary calculi using vNCT generated by DECT urogram compared to tNCT as a gold standard measure. The pooled sensitivity for vNCT for all studies was 78.1% (95% CI: 70.2 to 85%) with a very high overall heterogeneity *I*^2^ index of 92% (*p* < 0.001), suggesting the presence of significant differences in the sensitivity between studies.

Five subgroups resulted from the combined method variation groupings of oral hydration (500 to 1000 ml), use of IV furosemide and slice thickness or increment (≥ 2 mm and < 2 mm), known to potentially impact urinary calculus detection [9–11]. The subgroup of IV hydration was excluded from analysis as no studies met the inclusion criteria of > 250 ml of saline as described in the reference article [9]. Pooled estimates for urinary calculus diagnosis sensitivity for each of the subgroups are also displayed in Fig. 2.

The heterogeneity *I*^2^ index was suggestive of true underlying sensitivity differences between subgroups (*I*^2^ 96%, *p* < 0.001). However, as three subgroups contained only two studies, this estimate should be interpreted with caution.

The subgroup of vNCT for diagnosis of urinary calculi on studies that used no oral hydration and slice thickness or increment of < 2 mm included three studies with a pooled sensitivity of 75.2% (95% CI: 64.4 to 84.5%); the heterogeneity *I*^2^ index was 80.8% (*p* < 0.0001).

The subgroup of four studies that used oral hydration and a slice thickness or increment of < 2 mm reported the highest pooled sensitivity for the detection of urinary calculi using vNCT of 92.2% (95% CI: 89.5 to 94.5%) with no evidence of heterogeneity between studies (*I*^2^ 14.3% (*p* = 0.30)).

The reported sensitivity for the subgroup vNCT for diagnosis of urinary calculi on studies that used no oral hydration and a slice thickness or increment of > 2 mm was the lowest of all subgroups with pooled sensitivity of 58.5% (95% CI: 49 to 67.7%). There was a large difference in reported sensitivities between the two studies in this subgroup. The funnel plot to investigate potential publication bias (Appendix 2) demonstrated both studies in this subgroup were small and more prone to small study bias compared to other subgroups.

Two studies used no oral hydration, slice thickness or increment > 2 mm, and IV furosemide. The pooled sensitivity of this subgroup was 79% (95% CI: 72.7 to 84.6%). The subgroup of two studies that used oral hydration, slice thickness or increment of > 2 mm, and IV furosemide reported a pooled sensitivity of 67% (95% CI: 60.5 to 73.2%).

## Discussion

In this systematic review and meta-analysis, vNCT generated from DECT demonstrated a moderate pooled sensitivity of 78.1%. High heterogeneity was demonstrated between studies partially due to variation between study methods. Subgroups for methodology differences account for some of the heterogeneity between studies. The subgroup of oral hydration and slice thickness or increment of < 2 mm recorded the highest sensitivity of 92.2%. These findings are of importance in suggesting the potential of replacing the tNCT phase in CT urography split-bolus protocol with vNCT for the detection of urinary calculi.

A vNCT DECT urography protocol resulted in increased efficiency with reduced time on the CT scanner [19–21]. Significant reductions in radiation dose (28 to 47%) to the patients undergoing CT urography, the gold standard imaging method for haematuria screening [2, 8, 24].

The subgroup of studies that used < 2 mm slice thickness or increments had the highest and third-highest pooled sensitivity of the five subgroups. Keteslegers et al [11] study demonstrated that thinner slice thickness improved sensitivity for detecting urinary calculi by reducing partial volume effects on tNCT. Four out of six studies that used slice thickness or increment ≥ 2 mm reported the size of the undetected urinary calculi as significantly smaller than the calculi that were detected (Appendix 2). vNCT may also be further affected by over- or under-subtraction of iodine contrast that may mask calculi secondary to partial voluming.

While the usual aim of excretory phase imaging in CT urography is to opacify the entire renal collecting systems with contrast, this can pose challenges for vNCT generation. Iodine subtraction became less accurate when urine densities exceeded 740 HU [29].

Oral hydration improved sensitivity of urinary calculi on vNCT by diluting the concentration of iodine excreted by the kidneys into the urinary tract by inducing mild diuresis [9]. Weatherspoon et al [10] compared oral hydration and IV hydration for CT urography and concluded there was no significant difference in the ability to dilute iodine concentration in the ureters. Oral hydration was therefore identified as the superior choice as it was more cost effective and required less resources.

Silverman et al [9] hypothesised that IV furosemide increased the concentration of iodine within urine by increasing the urine flow rate in all segments of the renal tract. This resulted in an increased opacification of the ureters. An increase in iodine concentration in the urinary collecting system resulted in a decrease in sensitivity of the vNCT for the excretory phase of the DECT urogram [26].

One of the limitations of this meta-analysis was the heterogeneity between studies with a high calculated inconsistency (*I*^2^) of 92% overall [26]. Heterogeneity between subgroups was high but lower within the subgroups themselves where heterogeneity could be calculated. Heterogeneity could only be calculated if there were more than two studies. Heterogeneity was contributed to by the high degree of variation in DECT urogram protocols reported in these studies (Appendix 1).

Published sensitivity values were relied upon as there was no access to raw data for included studies. A limitation of this study was that pooling of specificity data and receiver operating characteristic (ROC) curves could not be performed due to a lack of available data reported (Table 4). With sensitivity calculated at the per-calculus level, as in these studies, estimates were likely biased due to unaccounted for correction between multiple calculi within a patient or segment. Without the provision of sensitivity at the patient level having accounted for corrections due to multiple calculi, the true diagnostic accuracy cannot be calculated [27]. It is recommended that further study be performed that specifically addresses this lack of data to give a more accurate picture of the diagnostic accuracy of vNCT in the detection of urinary calculi.

The use of tNCT as the gold standard for detecting renal tract calculi was employed in all studies due to the high reported sensitivity of this examination. Operational confirmation was not feasible, and as such, reported tNCT TP and FP values were assumed to be correct.

A further limitation of this meta-analysis was the exclusion of non-English-language studies, which may have resulted in a publication bias. Publication bias was further investigated using a funnel plot that identified potential small study bias (Appendix 3). The QUADAS tool assessment identified potential or unclear risk of bias in the research methodology which could affect the calculated sensitivity.

Only one of the thirteen studies utilised rapid-switching DECT scanners rather than dual-source DECT [17]. There were no articles included that used detector-based DECT scanners. This was due to the lack of any studies investigating the detection of urinary calculi using this technologies. The ability of detector-based DECT to use material decomposition to generate accurate vNCT has been described in other anatomical structures [28].

## Conclusion

While the overall sensitivity of the pooled data from the excretory phase of the DECT urography was moderate, promising subgroup protocols have been identified. When employing oral hydration and < 2 mm slice thickness and increment, a higher sensitivity was observed. Further research may allow incorporation of the vNCT technique and thus reduce radiation exposure to the patient, time on the CT scanner, and improved efficiency.

## Appendix

Appendix 1. Summary of technical parameters for included studies (DOCX 23 kb) Appendix 2. Reported urinary calculi size for detected and undetected calculi by vNCT (DOCX 21 kb) Appendix 3. Funnel Plot of sensitivity by subgroup. (JPG 102 kb)

## Abbreviations

CI: Confidence interval
CT: Computed tomography
DE: Dual energy
DECT: Dual-energy computed tomography
FDA: U.S. Food and Drug administration
FN: False negative
FP: False positive
IV: Intravenous
MDCT: Multidetector computed tomography
mm: Millimetres
PRISMA: Preferred Reporting Items for Systematic reviews and Meta-Analyses
QUADAS: Quality assessment of diagnostic accuracy studies
TN: True negative
tNCT: True non-contrast computed tomography
TP: True positive
vNCT: Virtual non-contrast computed tomography

## Declarations

### Guarantor

The scientific guarantor of this publication is Katherine McCoombe.

### Statistics and biometry

One of the authors has significant statistical expertise.

### Methodology

• Retrospective

• Systematic review and meta-analysis

• Performed at one institution
