# Supplementary material for: Sensitivity of virtual non-contrast dual-energy CT urogram for detection of urinary calculi: a systematic review and meta-analysis
Source: Eur Radiol. 2022 Jun 28;32(12):8588–96. doi: 10.1007/s00330-022-08939-5 (PMC9705483; doi:10.1007/s00330-022-08939-5)
Supplement: Appendix 1. — Summary of technical parameters for included studies (DOCX 23 kb) [file 330_2022_8939_MOESM1_ESM.docx]

|  | | | | | | | |
| --- | --- | --- | --- | --- | --- | --- | --- |
| **Appendix 1. Summary of technical parameters for included studies** | | | | | | | |
| **Literature**  **(author and year)** | **Technique** | | | | | | |
|  | **CT vendor/model** | **Dual-energy technique** | **IV Contrast type, dose, and injection rate** | **Scan delay for dual-energy acquisition** | **Urinary Dilution Technique** | **Non-contrast technique used** | **Slice Thickness x Increment** |
| Botsikas et al. 2014^24^ | Siemens SOMATOM Definition Flash (Siemens Medical Solutions) | Dual Source (tube A:100 kVp, tube B: 140 kVp, reference 250 mAs) | 1. 1.2 ml/kg @ 2ml/s (Accupaque 350; GE Healthcare)   0.8 ml/kg @ 2ml/s (Accupaque 350; GE Healthcare) | 1. 7 min between first and second contrast bolus   Scan performed 100s after second contrast bolus | 20 mg furosemide IV (Lasix, Sanofi Aventis) injected 1 min prior to first contrast bolus | Dual Source (tube A:100kVp, tube B: 140 kVp, reference 250 mAs) | 2 mm x 2 mm |
| Chen et al. 2015^16^ | Siemens SOMATOM Definition Flash (Siemens Medical Solutions) | Dual Source (tube A: 80 kVp, 300 mAs, tube B 140 kVp, 116 mAs) | 1. 50ml @ 2.5 ml/s (iopromide, 300 mgI/ml: Ultravist 300 Bayer Schering Pharma) 2. 70ml @ 2.5 ml/s (iopromide, 300 mgI/ml: Ultravist 300 Bayer Schering Pharma) | 1. 6 min between first and second contrast bolus   Scan performed 60 s after second contrast bolus | 1. 800 mls of water 20 min prior to scan 2. 200mls IV normal saline (2ml/s) prior to first contrast bolus 3. 25mls IV normal saine (2ml/s) post second contrast bolus | Single energy 120 kVp, 250 mAs | 1.5 mm x 1 mm |
| Chen et al. 2016^17^ | Siemens SOMATOM Definition Flash (Siemens Medical Solutions) | Dual Source (tube A: 80 kVp, 300 mAs, tube B: 140 kVp, 116 mAs) | 1. 0.8 ml/kg @ 2.5 ml/s (iopromide, 300 mgI/ml: Ultravist 300 Bayer Schering Pharma) 2. 1.2 ml/kg @ 2.5 ml/s (iopromide, 300 mgI/ml: Ultravist 300 Bayer Schering Pharma) | 1. 6 min between first and second contrast bolus   Scan performed 60 s after second contrast bolus | 1. 800 mls of water 20 min prior to scan 2. 200mls IV normal saline (2ml/s) prior to first contrast bolus   25mls IV normal saine (2ml/s) post second contrast bolus | Single energy 120 kVp, 250 mAs | 1.5 mm x 1 mm |
| Karlo et al. 2013^8^ | Siemens SOMATOM Definition Flash (Siemens Medical Solutions) | Dual Source (tube A:100 kVp, 210 mAs, tube B: 140 kVp, 160 mAs) | 1. 30 ml @ 3.5 ml/s (Iopromide, 300 mgI/ml: Ultravist 300 Bayer Schering Pharma) 2. 50ml @ 3.5 ml/s (iopromide, 300 mgI/ml: Ultravist 300 Bayer Schering Pharma) | 1. 9 min between first and second contrast bolus   Scan performed 90s after second contrast bolus | 1. 40 ml normal saline post firsts contrast bolus 2. 40mls normal saline post second contrast bolus | Single energy 120 kVp, 100 mAs | 2 mm x 1.6 mm |
| Lv et al. 2014^25^ | Discovery CT750 HD system (GE Healthcare) | Fast kVp switching between 80 and 140 kVp (550-640 mAs).  WB MD (water-iodine pair) and CaB MD (calcium-iodine pair) images reconstructed from single spectral CT acquisition | 1.5ml/kg @ 3-4ml/s (Optiray, 320 mg/ml, Tyco Health-care) | Renocortical phase – 12s post trigger attenuation threshold (100HU)  Nephric phase – 30 sec after renocortical  Excretory phase – 30 min post contrast | 40s IV normal saline (3-4 ml/s) post contrast bolus | Helical mode 120 kVp (227-346 mAs) | 0.625 mm x 0.625 mm |
| Mangold et al. 2012^15^ | Siemens SOMATOM Definition Flash (Siemens Medical Solutions) | Dual Source (tube A:100 kVp 115 mAs, tube B: 140 kVp 89 mAs) | 100 ml @ 2ml/s (Optiray, 300 mg/ml, Tyco Health-care) | 10 min | 50 ml normal saline post contrast bolus | Single energy 120 kVp, 210 mAs | 2 mm x 1 mm |
| Manoharan et al. 2020^2^ | Siemens SOMATOM Definition Flash (Siemens Medical Solutions) | Dual Source (tube A:100 kVp, 210 mAs tube B: 140 kVp, 160 mAs) | 1. 0.8 ml/kg @ 2ml/s (300 mgI/ml; Omnipaque, GE Healthcare)   1 ml/kg @ 4 ml/s (300 mgI/ml; Omnipaque, GE Healthcare) | 1. 8 min between first and second contrast bolus   Scan performed 60 s after second contrast bolus | 1. 1000 ml water 30 min prior to scan 2. 100 ml normal saline 2 ml/s post first contrast bolus 3. 30 ml normal saline 4 ml/s post second contrast bolus | Dual energy (tube A:100kVp, 210 mAs tube B: 140 kVp, 160 mAs) | 1.5 mm x 1.5 mm |
| Moon et al. 2012^20^ | Siemens SOMATOM Definition Flash (Siemens Medical Solutions) | Dual Source (tube A: 80 kVp, 404 mAs, tube B: 140 kVp, 96 mAs) | 120 ml @ 3 ml/s (Ultravist 300 Bayer Schering Pharma) | 1. 100s (nephric)   8 min or 12 min (excretory) | Prior to 8min excretory phase furosemide. Or no furosemide given when excretory phase done 12 post contrast injection. | Single energy 120 kVp, 240 mAs | 1.5 mm x 3 mm |
| Park et al. 2016^19^ | Siemens SOMATOM Definition Flash (Siemens Medical Solutions) | Dual Source (tube A: 140 kVp, 96 mAs, tube B: 80 kVp, 404 mAs) | 120mls @ 3ml/s (Ultravist 300 Bayer Schering Pharma) | 1. 80-100s (nephric)   8 min (excretory) | 1. 1000 ml water 30 min prior to scan   0.1 mg/kg IV furosemide injected prior to contrast | Single energy 120 kVp, 240 mAs | 1.5 mm x 3 mm |
| Sahni et al. 2013^18^ | Siemens Definition (Siemens Medical Solutions) | Dual Source (tube A: 80 kVp, 340 mAs, tube B: 140 kVp, 80 mAs,) | 80 ml (Ultravist 370 Bayer Healthcare Pharmaceuticals) | 1. 100s (nephric)   15 min (excretory) | 1. 900ml water orally   10 mg IV furosemide (Lasix; Abbott Laboratories) injected 2-3 min prior to contrast bolus or 250mls of IV normal saline post contrast | Dual Source (tube A: 140 kVp, 80 mAs, tube B: 80 kVp, 340 mAs) | 3 mm x 3 mm |
| Takahashi et al. 2013^26^ | Siemens SOMATOM Definition Flash (Siemens Medical Solutions) | Dual Source (tube A: 80 kVp, 340 mAs tube B: 140 kVp, 80 mAs,) | 1. 50 ml @ 4ml/s (300mg iodion/ml; omnipaque, GE Healthcare)   90 ml @ 4ml/s (300mg iodion/ml; omnipaque, GE Healthcare) | 1. 10 min delay between first and second contrast bolus 2. Scan performed 90 sec after second contrast bolus | 200mls IV normal saline (4ml/s) prior to first contrast bolus  Abdominal compression used for 60 patients | Dual Source (tube A: 140 kVp, 80 mAs, tube B: 80 kVp, 340 mAs) | 1.5 mm x 1.5 mm |
| Toepker et al. 2014^22^ | Siemens SOMATOM Definition Flash (Siemens Medical Solutions) | Dual energy (tube A: 80 kVp, 233 mAs, tube B: 140 kVp, 180 mAs) | 1. 15 ml Iomeron (400mg/ml)   80 ml Iomeron (400mg/ml) | 1. 10 min between first and second contrast bolus   Scan performed 65s after second contrast bolus | None | Single energy (120 kVp, 200 mAs) | 1 mm x 0.8 mm |
| Yeo et al. 2015^21^ | Siemens SOMATOM Definition Flash (Siemens Medical Solutions) | Dual Source scanner  120 kVp 100 mAs (patients >80 kg)  100 kVp 210/160 mAs (patients <60kg) | 1. 50 ml @ 2 ml/s (iopromide, 300 mgI/ml: Ultravist 300 Bayer Schering Pharma) 2. 100ml @ 2ml/s (iopromide, 300 mgI/ml: Ultravist 300 Bayer Schering Pharma) | 1. 9 min between first and second contrast bolus   Scan performed 100s after second contrast bolus | 1. 500 ml of water 30 min prior to scan 2. 40s IV normal saline (2ml/s) post first contrast bolus   40s IV normal saline (2ml/s) post second contrast bolus | Dual-energy 120 kVp 100 mAs (patients >80kg)  100 kVp 210/160 mAs (patients <60kg) | 1.5 mm x 1.5 mm |
