# Supplementary material for: Sensitivity of virtual non-contrast dual-energy CT urogram for detection of urinary calculi: a systematic review and meta-analysis
Source: Eur Radiol. 2022 Jun 28;32(12):8588–96. doi: 10.1007/s00330-022-08939-5 (PMC9705483; doi:10.1007/s00330-022-08939-5)
Supplement: Appendix 2. — Reported urinary calculi size for detected and undetected calculi by vNCT (DOCX 21 kb) [file 330_2022_8939_MOESM2_ESM.docx]

| **Appendix 2. Reported urinary calculus size for detected and undetected calculus by vNCT** | | | | |
| --- | --- | --- | --- | --- |
| Study | Number of Undetected Calculi | Detected Calculus size  Mean ± SD | Undetected Calculus size  Mean ± SD | P Value |
| Botsikas et al. 2014^24^ | 5 | 7.3±3.2mm | ≤2mm | NA |
| Chen et al. 2015^16^ | 4 | 0.17±0.47cm² | Less than 0.03cm² | <0.05 |
| Chen et al. 2016^17^ | 6 | 0.35±0.84 cm² | Smaller than 0.03cm² | <0.05 |
| Karlo et al. 2013^8^ | 18 | 5mm (2-27mm) | 2.5 mm (1-4mm) | <0.001 |
| Lv et al. 2014^25^ | 35 | 7.91±8.11mm | NA | 0.083 |
| Mangold et al. 2012^23^ | 41 | 5±3.6mm | NA | 0.005 |
| Manoharan et al. 2020^2^ | 11 | 7.11±5.78 mm | 2mm (1-6.4) | 0.969 |
| Moon et al. 2012^20^ | 21 | 5.2±3.6mm | 2.2±0.4mm | <0.0001 |
| Park et al. 2016^19^ | 48 | 5.2±3.6 mm | 1.6±0.6mm | <0.001 |
| Sahni et al. 2013^18^ | 22 | NA | 2.1 (1-4mm) | NA |
| Takahashi et al. 2013^26^ | 16 | NA | NA | NA |
| Toepker et al. 2014^22^ | 61 | NA | NA | NA |
| Yeo et al. 2015^21^ | 38 | 3.6 (1-22mm) | NA | 0.01 |
| *Median (IQR) | | | | |
