# Supplementary figures and images for: Sensitivity of virtual non-contrast dual-energy CT urogram for detection of urinary calculi: a systematic review and meta-analysis
Source: Eur Radiol. 2022 Jun 28;32(12):8588–96. doi: 10.1007/s00330-022-08939-5 (PMC9705483; doi:10.1007/s00330-022-08939-5)

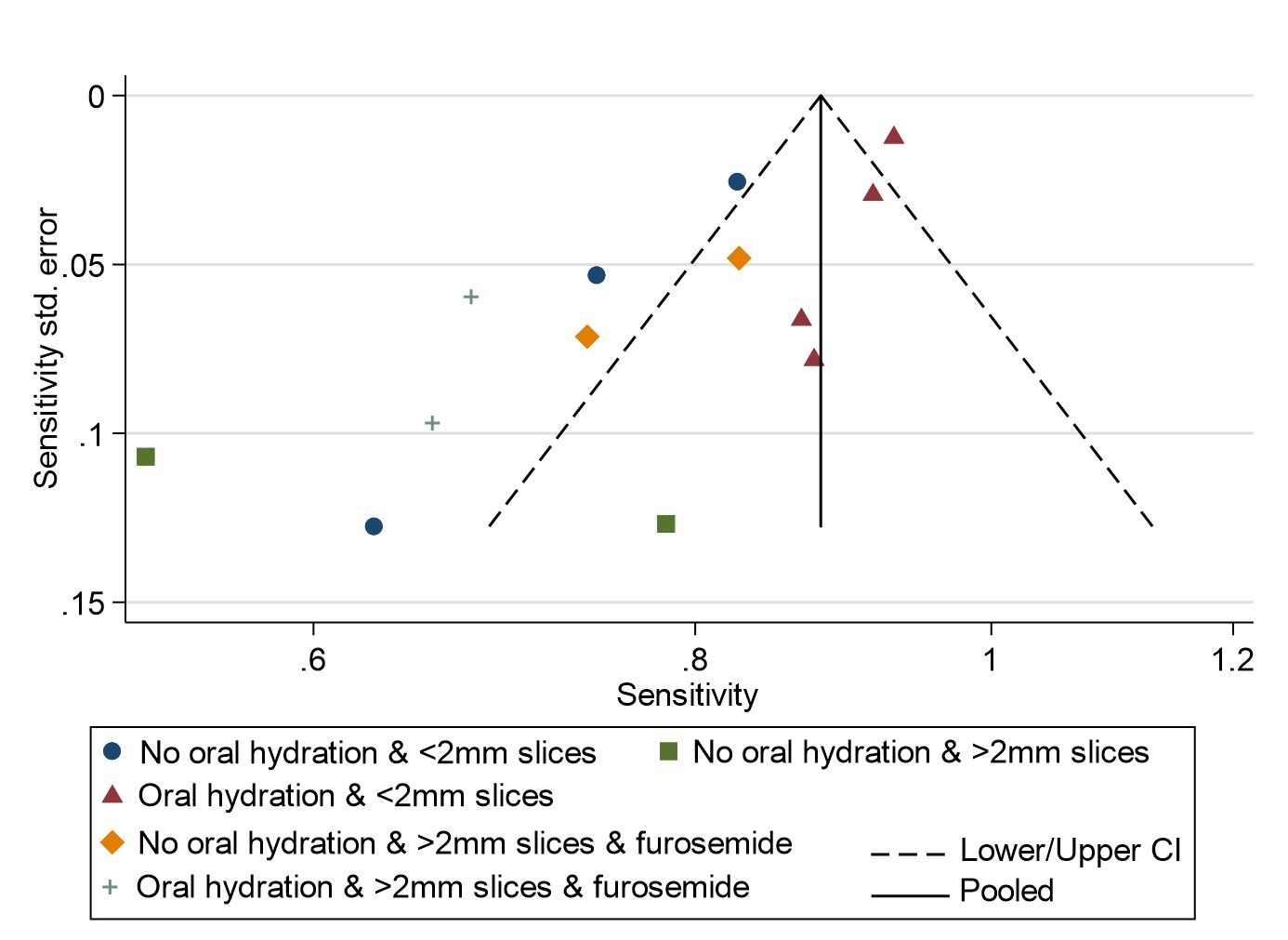

Supplement: Appendix 3. — Funnel Plot of sensitivity by subgroup. (JPG 102 kb) [file 330_2022_8939_MOESM3_ESM.jpg]
